# Two-Step Enzymolysis of Antarctic Krill for Simultaneous Preparation of Value-Added Oil and Enzymolysate

**DOI:** 10.3390/md21010047

**Published:** 2023-01-11

**Authors:** Xin-Nan Teng, Shu-Chang Wang, Liaqat Zeb, Yue-Sheng Dong, Zhi-Long Xiu

**Affiliations:** School of Bioengineering, Dalian University of Technology, Dalian 116024, China

**Keywords:** Antarctic krill oil, enzymolysis, protease, chitinase, extraction

## Abstract

Antarctic krill is a crucial marine resource containing plenty of high-valued nutrients. However, krill oil as a single product has been developed by the current solvent extraction with high cost. From the perspective of comprehensive utilization of Antarctic krill, this study proposed a novel two-step enzymolysis-assisted extraction in attempt to produce value-added oil and enzymolysate simultaneously. After two-step chitinase/protease hydrolysis, the lipid yield increased from 2.09% to 4.18%, reaching 112% of Soxhlet extraction. The method greatly improved the yields of main components while reducing the impurity content without further refining. After optimization, the oil contained 246.05 mg/g of phospholipid, 80.96 mg/g of free eicosapentaenoic acid (EPA) and docosahexaenoic acid (DHA), and 0.82 mg/g of astaxanthin. The by-product enzymolysate was abundant in water-soluble proteins (34.35 mg/g), oligopeptides (13.92 mg/g), amino acids (34.24 mg/g), and carbohydrates (5.79 mg/g), which was a good source of functional nutrients. In addition, both oil and enzymolysate showed high antioxidant capacity. This novel method could simultaneously provide oil and enzymolysate amounting for 58.61% of dried krill.

## 1. Introduction

Antarctic krill (*Euphausia superba*), a species living in the Antarctic Ocean, is of great importance for many mammals and fish as food. Because of abundance in proteins, lipids and carbohydrates, Antarctic krill has gradually become a dominant source of proteins and fats for human beings due to the increasingly depleting fish resources [1]. Fresh Antarctic krill contains approximately 0.5~3.6% of lipid, of which the polar lipid represented by phospholipid (PL) is the highest in concentration, followed by triglyceride, cholesterol and free fatty acid (FFA) [2,3]. Antarctic krill oil (AKO) is rich in ω-3 polyunsaturated fatty acids (PUFAs) such as eicosapentaenoic acid (EPA) and docosahexaenoic acid (DHA), and contains 30~65% of lipids in the form of PLs [3,4]. In addition, astaxanthin, a natural strong antioxidant, is also present in AKO [5]. Containing plenty and variety of nutrients, AKO possesses a lot of health benefits, including neuroprotection, improvement of alcoholic liver injury, inhibition of obesity, diabetes, and various cancers [6,7,8,9,10,11]. In particular, the excellent antioxidant capacity of AKO contributed by numerous direct or indirect antioxidants has been proved to be the basis of many bioactivities [12,13,14].

The yield, composition and activity of AKO are greatly affected by the extraction methods [15]. Considering the high safety, low equipment requirements and a plethora of active components, the preferred extraction method of AKO in food industry is solvent extraction with ethanol as the solvent [10]. However, the lipid-rich areas in Antarctic krill are along the digestive tract, between muscle bundles and beneath the exoskeleton [16]. The solvent cannot penetrate through the krill effectually and extract the oil adequately due to the presence of chitin and structure protein. In addition, compared with hydrophobic organic solvents such as n-hexane and acetone, the AKO extracted by high polar ethanol contains more soluble impurities, which reduce the product quality and value.

The enzymolysis-assisted extraction, which can release more oil based on the destruction of the raw material structure, is now an emerging lipid extraction technology. At present, the main enzymes used for lipid extraction are all proteases [17]. Among them, alkaline protease and compound protease have been proved to improve the yield of AKO significantly [18,19]. However, all the protease-based enzymolysis-assisted extractions are improving the oil release by hydrolyzing the structure proteins in raw materials, and the restriction of krill chitin exoskeleton on the AKO release has not been solved. Chitinase, which splits β-1,4 glycosidic bonds in chitin into chitin oligosaccharides and N-acetyl-D-glucosamine, is widely present in fungi, bacteria, arthropods and plants [20]. Chitinase is mainly used as food preservative or to produce chitin oligosaccharide or N-acetylglucosamine as biologicals or fermentation substrates in the food industry [21]. Few studies on assisting lipid extraction were reported.

In this study, chitinase and protease were used separately, simultaneously and successively in different enzymolysis methods before ethanol extraction. The effects of enzymolysis on lipid yield and quality were analyzed. Accordingly, a novel two-step enzymolysis-assisted extraction was established. Then, the effect of hydrolysis time was investigated. Finally, the composition and content of active components in AKO and enzymolysate under the optimal conditions as well as their antioxidant capacities were further analyzed. This study will provide a new approach for the extraction and refining of value-added AKO and enzymolysate in the food industry.

## 2. Results and Discussion

### 2.1. Effect of Enzymolysis Method on Antarctic Krill Oil Yield

**Figure 1:**
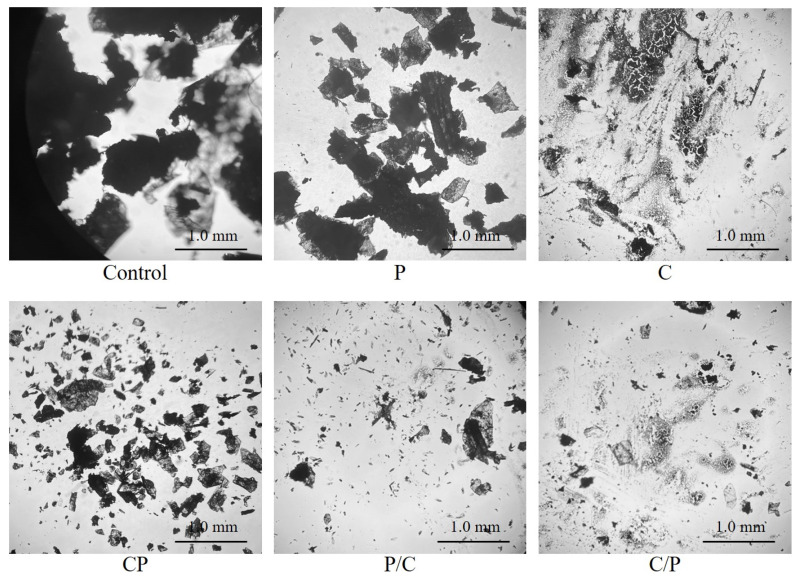
Micrograph of Antarctic krill residuals after enzymolysis. P: single protease hydrolysis; C: single chitinase hydrolysis; CP: simultaneous hydrolysis using protease and chitinase; P/C: two-step hydrolysis using protease followed by chitinase; C/P: two-step hydrolysis using chitinase followed by protease. Scale bar represents 1.0 mm.

Single chitinase, protease and their different combinations were used to pretreat krill before ethanol extraction. Figure 1 shows the morphology of Antarctic krill residuals obtained after enzymolysis. The micrograph of homogenized krill materials (control) shows a clear structure frame composed of proteins and chitin. However, the krill structure was destructed in different forms after single or complex enzyme hydrolysis. For single alkaline protease pretreatment (group P), the thickness of the fragments decreased significantly due to the reduction in structure proteins, although the chitin exoskeleton of hydrolyzed krill still presented a relatively complete morphology. In contrast, after hydrolysis by single chitinase (group C), the obvious increase in the fragmentation degree indicated the destruction of chitin skeleton, while the small structure pieces indicated the existence of the protein structure. For the krill residue after simultaneous hydrolysis by means of alkaline protease and chitinase (group CP), similar results to the single protease treatment (group P) were observed, and the degree of fragmentation was higher. The morphology of krill residues after two-step hydrolysis (group P/C and C/P) showed that two-step hydrolysis achieved a good effect of destroying both krill structure protein and chitin skeleton. Comparatively, the effect of using chitinase followed by protease (group C/P) performed better.

**Figure 2:**
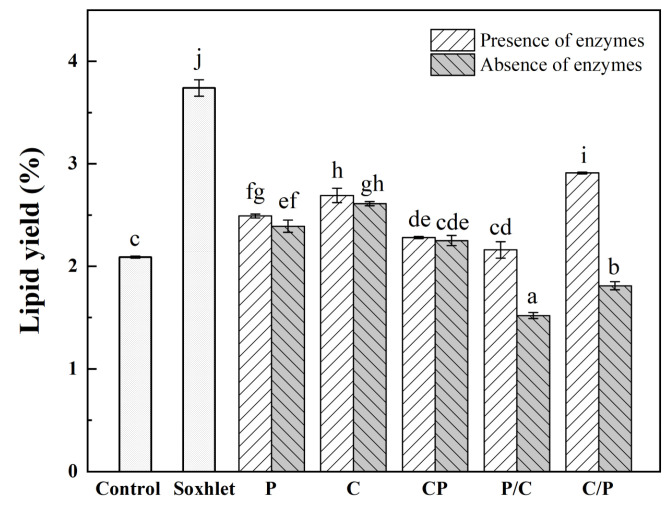
Antarctic krill oil yield of different extraction methods. P: single protease hydrolysis; C: single chitinase hydrolysis; CP: simultaneous hydrolysis using protease and chitinase; P/C: two-step hydrolysis using protease followed by chitinase; C/P: two-step hydrolysis using chitinase followed by protease. Different letters on the top of data indicate significant differences (Duncan’s test, *p* < 0.05).

The lipid yields of the various enzymolysis-assisted extractions are presented in Figure 2. The results showed that the lipid yield of AKO varied from 2.16% to 2.91% in the presence of enzymes, which were higher than 2.09% of the control. Specifically, the highest yield was obtained in the two-step hydrolysis group using chitinase followed by alkaline protease (group C/P), i.e., 1.4 times that of the control. The simultaneous hydrolysis (group CP) and the two-step hydrolysis using protease followed by chitinase (group P/C) were relatively low compared with the single alkaline protease (group P) and single chitinase hydrolysis (group C). Compared with the two-step hydrolysis in group C/P, the oil yield obtained by simultaneous hydrolysis (group CP) was lower in spite of two types of enzymes. This was owing to the enzymolysis conditions (pH and temperature) not meeting the optimal conditions of either enzyme simultaneously, causing a low hydrolysis efficiency. Moreover, the hydrolysis of chitinase by protease further reduced the effect of enzymolysis. Interestingly, no significant difference in the lipid yield occurred between the groups with or without enzymes despite single enzyme (group P and C) or simultaneous hydrolysis (group CP). This was attributed to the existence of endogenous enzymes in the raw materials without heating or drying, e.g., lipase, protease and chitinase, which would also function under suitable conditions [22]. However, in the two-step hydrolysis groups (P/C and C/P), the endogenous lipase could hydrolyze more fat to produce FFAs in a longer enzymolysis time, resulting in lower lipid yield than that of groups C, P, and CP in the absence of added enzymes. The addition of protease would hydrolyze lipase and other endogenous enzymes as well as the added chitinase. This might be the reason for a lower yield achieved in group P/C than that of group C/P in the presence of added enzymes. The lipid yield of Soxhlet extraction with ethanol as solvent was 3.74%, which was significantly higher than that of the control group and 1.29 times higher than that of the two-step hydrolysis groups, while the enzymolysis conditions needed to be further optimized. These results indicated that the two-step enzymolysis-assisted extraction may be a good approach for producing AKO in terms of lipid extraction efficiency.

A few studies on the protease-assisted extraction of AKO were reported. For example, the effects of six proteases (papain, compound protease, acidic protease, neutrase, pancreatin and alcalase) were investigated on the lipid yield and quality of AKO [18]. Compared with the solvent extraction, the quality and lipid yield of krill oil could be improved by enzymolysis, and the best result was performed by alkaline protease. Therefore, in this study, alkaline protease was selected as one of the enzymes in the two-step enzymolysis. Nevertheless, chitinase-assisted oil extraction has rarely been reported so far. This enzyme was mainly used for chitin oligosaccharides or N-acetyl-D-glucosamine production. For example, N,N′-diacetylchitobiose and N-acetyl-D-glucosamine were obtained from the defatted and deproteinized Antarctic krill residues by the aid of a cocktail of chitinolytic enzymes [23]. In this study, we used chitinase to extract AKO with high yield, and the results showed that even single chitinase could hydrolyze the chitinaceous skeleton of Antarctic krill and release more oil. Based on chitinase followed by alkaline protease, the two-step enzymolysis-assisted extraction could achieve a better extraction efficiency than that of single enzyme (chitinase or protease).

### 2.2. Effect of Enzymolysis Method on the Antarctic Krill Oil Quality

**Figure 3:**
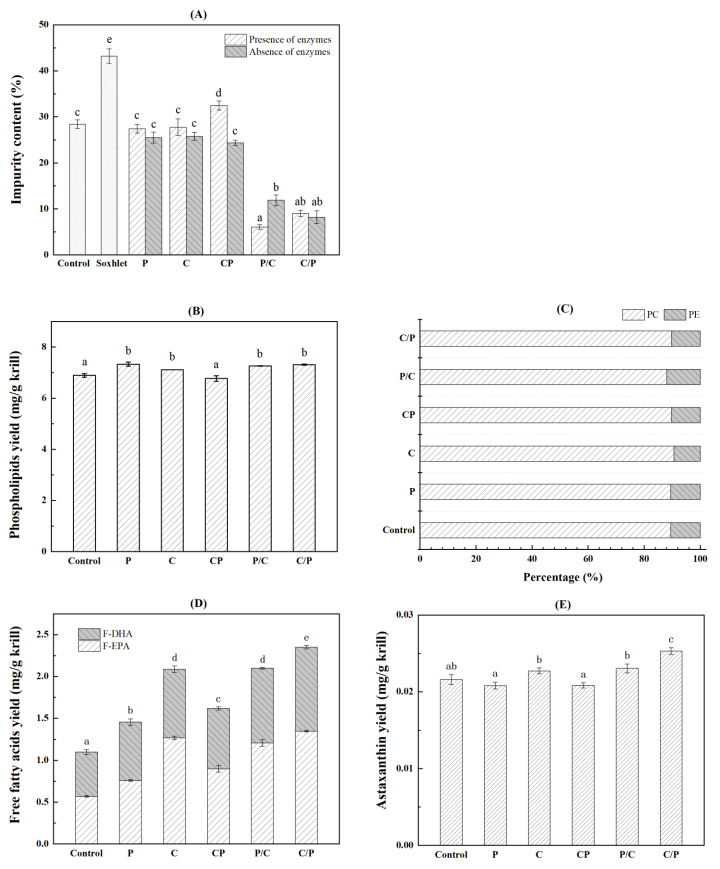
Comparison of impurity content (**A**), phospholipids yield (**B**) and composition (**C**), free fatty acid yield (**D**) and astaxanthin yield (**E**) among different enzymolysis-assisted extraction methods. P: single protease hydrolysis; C: single chitinase hydrolysis; CP: simultaneous hydrolysis using protease and chitinase; P/C: two-step hydrolysis using protease followed by chitinase; C/P: two-step hydrolysis using chitinase followed by protease. Abbreviations: PC, phosphatidylcholine; PE, phosphatidyl ethanolamine; F-DHA, free docosahexaenoic acid; F-EPA, free eicosapentaenoic acid. Different superscript letters indicate significant differences (Duncan’s test, *p* < 0.05).

After ethanol extraction, crude oil was obtained by low-speed centrifugation of the oil-rich solvent phase, and the refined AKO was further obtained by a secondary high-speed centrifugation of crude oil. The impurity separated from crude oil was different as shown in Figure 3A. Compared with the impurity content of 28.39% in the control group, the hydrolysis by single enzyme (group P or C) or two enzymes (group CP) could not decrease the impurity content in crude oil significantly; simultaneous hydrolysis even increased to a certain extent. However, the two-step hydrolysis using two enzymes (groups P/C and C/P) had a significant reduction in the impurity content. The impurity content in the groups P/C and C/P was 6.08% and 9.01%, respectively, which was 78.58% and 68.26% lower than that in the control group. In the two-step enzymolysis, more large proteins or chitin were hydrolyzed into small ones, e.g., soluble peptides, oligosaccharides, N-acetyl-D-glucosamine and amino acids, and separated by centrifugation before solvent extraction. The impurity content in each group without added enzymes was consistent with the corresponding group with added enzymes, indicating the endogenous enzymes in krill also performed similar effects. In addition to enzymolysis, protein precipitation at their isoelectric points (PI) might be another reason. Because both acidic and alkaline buffers were used in the two-step enzymolysis groups (C/P and P/C), more precipitated proteins could be expected than single enzyme (group P and C) or simultaneous enzymes (group CP) hydrolysis at a certain pH. Similar results were reported for the isolation of muscle protein from Antarctic krill and silver carp using the isoelectric solubilization/precipitation [24,25]. Although the lipid yield of Soxhlet extraction was far ahead, its impurity content was also significantly higher than that of other groups. These results indicated that two-step enzymolysis could reduce the impurity content in the crude AKO effectively and avoid the additional process and cost for further refining.

Figure 3B shows the effect of enzymolysis on PL yield. In absence of adding enzymes, the PL yield was 6.89 mg/g krill in the control group. A similar PL yield was achieved in the group CP. Other groups (P, C, P/C, C/P) exhibited significantly different results from the control group. The PL yield obtained in the group C/P reached 7.33 mg/g krill, which was about 6% higher than that of control. These results were consistent with the alkaline protease hydrolysis reported by Wang et al. [18]. Figure 3C shows no significant difference in PL composition (phosphatidylcholine, PC and phosphatidyl ethanolamine, PE) among different enzymolysis modes. Specifically, as the main component of PL, PC accounts for 88.09%~90.62%, which is lower than the PC content of 95.16% obtained by Xie et al. [15]. It might be related to the source, harvest season, storage time and pretreatment method of Antarctic krill. PL is the most abundant active component in AKO. As a component of cell membrane with unique biocompatibility, PL is an important evaluation index of oil [4,26]. These results showed that the two-step chitinase/protease hydrolysis-assisted extraction could significantly improve the PL yield of AKO without changing the PL composition.

As shown in Figure 3D, various enzymolysis modes exerted great influence on the yield of F-EPA and F-DHA. The total yield of F-EPA and F-DHA increased by 116% from 1.09 mg/g krill in the control group to 2.35 mg/g krill in the group C/P. EPA and DHA are characteristic and crucial components of marine oils. Owing to the presence of highly active lipases in the digestive glands of Antarctic krill, autolysis and subsequent rapid degradation of lipids often occurs in fishing, storage and processing, resulting in the increase in FFA content [27]. For ordinary edible oil, the existence of FFAs will increase acid value, reduce the oil quality and shorten the shelf life. However, the FFAs in AKO with about 30% of n-3 PUFA usually contain a large amount of F-EPA and F-DHA, which have unique nutritional value and bioavailability. Figure 3D shows that the total yields of F-EPA and F-DHA were significantly increased after chitinase hydrolysis, regardless of single chitinase hydrolysis or two-step hydrolysis, and especially for F-EPA. Compared with the PL yield in Figure 3B, chitinase hydrolysis could increase FFA yield without decline in PL yield, which might be related to the higher activity of triglyceride hydrolase and lower activity of phospholipase under the hydrolysis conditions of chitinase, i.e., pH 6.0 and 45 °C [27].

As the most important minor component in AKO, the yield of astaxanthin is shown in Figure 3E. Compared with control group (0.0216 mg/g krill), the astaxanthin yield showed no significant increase after single enzyme pretreatment, which was coincident with the alkaline protease hydrolysis reported by Wang et al. [18]. Similar results were shown in the group CP and P/C, while the astaxanthin yield increased significantly in the group C/P, reaching 0.0253 mg/g krill, which meant more astaxanthin was released during two-step enzymolysis using chitinase followed by protease.

Overall, the two-step chitinase/protease hydrolysis-assisted extraction could greatly improve the quality of AKO, i.e., the increased yields of FFA, astaxanthin, and PL without change in PL composition, while the reducing content of impurity in crude oil without further refining.

### 2.3. Screening of Two-Step Enzymolysis Conditions

**Figure 4:**
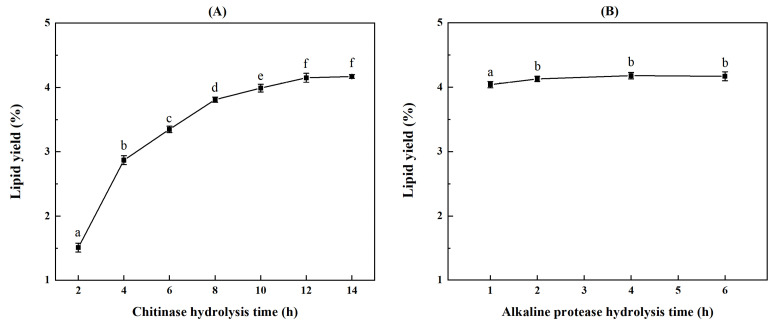
Effect of enzymolysis time on oil yield of Antarctic krill during two-step enzymolysis using chitinase followed by protease. (**A**) Chitinase hydrolysis time and (**B**) alkaline protease hydrolysis time. Different letters on the top of data indicate significant differences (Duncan’s test, *p* < 0.05).

The two-step chitinase/protease hydrolysis exhibited superior influence on the yield and quality of AKO. Based on the fact that the optimum pH and temperature of enzymolysis were chosen according to their characteristics provided by the manufacturers, the hydrolysis time would play an important role in the enzymolysis. Therefore, in order to improve the extraction yield, single-factor experiments were conducted to investigate the effect of enzymolysis time in two steps as shown in Figure 4. The AKO yield increased continuously as the prolongation of chitinase hydrolysis time until it reached a stable value of 4.15% at 12 h (Figure 4A). When the enzymolysis time of chitinase was fixed to 12 h, the effect of enzymolysis time of alkaline protease on oil yield was further investigated (Figure 4B). The lipid yield raised with the prolongation of time in the first 2 h, and then tended to be stable and decreased slightly with the increase in protease hydrolysis time. Chitinase hydrolysis was undoubtedly the key step in the two-step enzymolysis in terms of its contribution to improving the lipid yield. In this step, the shell and protein skeleton of Antarctic krill had been fully decomposed under the action of chitinase and endogenous enzymes. The first enzymolysis undoubtedly increased the oil release, and shortened the time of the second enzymolysis. Although the improved protease hydrolysis showed no obvious effect on the lipid yield, its contribution to reducing the impurity content in the crude oil (Figure 3A) and increasing the water-soluble proteins in enzymolysate was apparent. The final AKO yield obtained in two-step enzymolysis-assisted extraction was 4.18%, which was 112% and 200% of the Soxhlet extraction (3.74%) and the control group (2.09%), respectively. Apparently, the significant increase in lipid yield should be attributed to the two-step enzymolysis, leading to the obtained yield beyond the upper limit value reported by the solvent extraction (0.5~3.6%), although different raw materials also had a certain impact on the lipid content of krill [2]. This method could greatly improve the AKO yield, making it a crucial AKO extraction technology.

### 2.4. The Optimal Quality of Antarctic Krill Oil and Enzymolysate

The bioactivity of AKO is deeply affected by its composition and content of active components which are greatly influenced by extraction methods [28]. Thus, the active components of AKO extracted under the optimal conditions of two-step enzymolysis were analyzed (Table 1). Compared with the traditional ethanol extraction, the content of PL and astaxanthin in AKO after two-step chitinase/protease hydrolysis decreased slightly due to the significant increase in total lipid yield. Comparatively, the total FFA content in AKO increased significantly in the two-step enzymolysis extraction. In detail, the contents of F-EPA and F-DHA were increased by 87.22% and 48.51%, respectively, which would greatly improve the quality and bioavailability of AKO.

Except AKO, the enzymolysate of protease and chitinase was also produced as an important by-product by the two-step enzymolysis. Chitinase and protease were added successively to hydrolyze the insoluble chitins and proteins into soluble oligosaccharides, N-acetyl-D-glucosamine, peptides and amino acids, which were separated by centrifugation and retained in enzymolysate. As shown in Table 2, high contents of soluble proteins, polypeptides and amino acids were detected in the first and second enzymolysates. Owing to the existence of highly active proteases in the digestive gland of Antarctic krill, the protein components in the first enzymolysate were mainly derived from the hydrolysis of endogenous proteases during storage and the first-step enzymolysis. In addition, the protein components in the second enzymolysate were produced by the adding alkaline proteases. Moreover, compared with the second enzymolysate, the first enzymolysate contained higher polysaccharide content, which was contributed to the hydrolysis of chitin by the adding chitinase. No significant difference in reducing sugar content between the two enzymolysates showed that more oligosaccharides were released. Nowadays, more and more physiological activities of Antarctic krill peptides have been discovered, e.g., strong antioxidant activity and inhibition effect on diabetes and hypertension [29,30]. In addition, a large number of chitin oligosaccharides have been reported to possess outstanding anti-fungi, anti-tumor and anti-inflammatory activities [21]. Therefore, the by-product enzymolysate containing abundant active components could be a high-quality source of functional soluble polypeptides, amino acids and polysaccharides as nutritional and healthcare foods. After two-step enzymolysis-assisted extraction of Antarctic krill, the obtained oil and enzymolysate accounted for 18.83% and 39.78% of the initial krill weight in dry base, respectively, reaching total value-added utilization rate of 58.61%.

### 2.5. The Antioxidant Capacity of Antarctic Krill Oil and Enzymolysate

**Figure 5:**
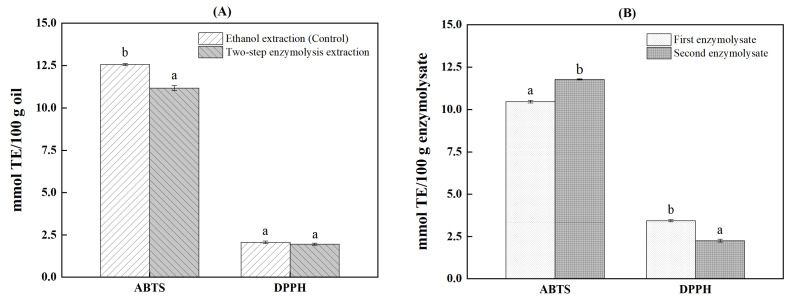
Free radical scavenging abilities of Antarctic krill oil and the enzymolysate extracted under the optimal conditions. (**A**) Antarctic krill oil and (**B**) enzymolysate. Different letters on the top of data indicate significant differences (independent samples *t* test, *p* < 0.05).

AKO is endowed with strong antioxidant capacity because of richness in astaxanthin, which exhibits a stronger antioxidant activity than vitamin E and β-carotene [31]. Meanwhile, as incremental krill peptides have been proven to have commendable antioxidant capacity, the peptide-rich hydrolysates are also considered to have remarkable antioxidative potential [29,32]. Therefore, DPPH• and ABTS•^+^ scavenging ability of AKO and the enzymolysate obtained under the optimal conditions for two-step enzymolysis were analyzed (Figure 5). As shown in Figure 5A, the two AKO samples showed strong free radical scavenging activities. Compared with the traditional ethanol extraction, the ABTS•^+^ scavenging ability of AKO obtained by two-step hydrolysis (11.17± 0.15 mmol TE/100 g oil) decreased significantly (11.07%), apparently due to the significant decrease in astaxanthin content. However, it was still much higher than that of AKO extracted by acetone–ethanol mixed solvents (<3 mmol TE/100 g oil) [33]. Comparatively, there was no significant difference in DPPH• inhibition between AKOs obtained by the two extraction methods. This phenomenon was due to astaxanthin having a strong absorption at the analytical wavelength of DPPH assay. The DPPH• scavenging ability of astaxanthin cannot be expressed accurately after the subtraction of background absorption [34]. Hence, the DPPH• scavenging abilities of both AKO samples (2.07 and 1.96 mmol TE/100 g oil) were significantly lower than that of ABTS•^+^ (12.56 and 11.17 mmol TE/100 g oil). This difference also suggested that in addition to astaxanthin, other components such as PL in AKO may play indirect antioxidant roles [1].

As shown in Figure 5B, the two enzymolysates showed high scavenging abilities on ABTS•^+^ and DPPH^•+^. Similar to AKO, the ABTS•^+^ inhibitions of the enzymolysates (10.45 and 11.77 mmol TE/100 g enzymolysate) were significantly higher than those of DPPH (3.44 and 2.26 mmol TE/100 g enzymolysates). Although both free radicals are N•, ABTS•^+^ (cationic free radical) scavenging is based on electron transfer (ET), while DPPH• (neutral free radical) inhibition is more contributed by hydrogen atom transfer (HAT) [32]. Apparently, the antioxidant activity of the enzymolysate was mainly attributed to ET. In addition, due to chitinase and protease being added successively, the compositions of the two enzymolysates were significantly different, which led to the distinction in scavenging ability of different free radicals. The two-step enzymolysis can retain most of the high antioxidant activity of AKO, and produce by-product enzymolysate with high antioxidant activity simultaneously.

## 3. Materials and Methods

### 3.1. Materials

Frozen Antarctic krill with a water content of 77.8 ± 2.8% (*w*/*w*) was provided by Liaoning Province Dalian Ocean Fishery Group Co., Ltd. (Dalian, China) and stored at −70 °C. PC, PE, EPA, DHA and astaxanthin standards, 2,2-diphenyl-1-picylhydrazyl (DPPH) and 2,2-azino-bis(3-ethylbenzthiazoline)-6-sulfonic acid (ABTS) were purchased from Shanghai Aladdin Biochemical Technology Co., Ltd. (Shanghai, China). Acetonitrile, methanol, n-hexane, and isopropanol (HPLC grade) were purchased from Sigma-Aldrich Co., Ltd. (Saint Louis, MO, USA). Alkaline protease (enzyme activity >200,000 U/g, optimal pH 8.5~10.5, optimal temperature 40~55 °C), bicinchoninic acid (BCA) protein assay kit, bovine serum albumin (BSA) and 6-hydroxy-2,5,7,8-tetramethylchroman-2-carboxylic acid (trolox) standards were purchased from Beijing Solarbio Science & Technology Co., Ltd. (Beijing, China). Food grade chitinase (enzyme activity 100,000 U/g, optimum PH 5~6, optimum temperature 45~60 °C) was purchased from Changmao Biochemical Engineering Co., Ltd. (Changzhou, China). Glucose standard and other reagents were purchased from Sinopharm Chemical Reagent Co., Ltd. (Beijing, China).

### 3.2. Enzymolysis

Krill samples were divided into 5 groups according to the single enzymolysis, simultaneous enzymolysis and two-step enzymolysis as well as the addition order of the two enzymes. The experiments were conducted by the modified method according to Wang et al. [18]: 20 g of smashed krill meal was weighed and homogenized with 20 mL 0.2 M phosphate buffer (alkaline protease pH 8.6, chitinase pH 6.0, and simultaneous hydrolysis pH 7.0). Then, 0.2 g of enzyme (0.2 g of each enzyme for simultaneous hydrolysis) was added. The enzymolysis was then carried out in a constant temperature oscillator (ZWYR-2102C, Zhicheng, Shanghai, China) at 45 °C for 4 h with a constant stirring at 200 r/min. After centrifugation (5920R, Eppendorf, Hamburg, Germany) at 3400× *g* for 20 min, the enzymolysate and sediment were separated for further analysis. For two-step enzymolysis, the sediment obtained from the first hydrolysis was introduced as substrate and the experiment was conducted referring to the first step. The enzymolysates were collected and dehydrated at 45 °C with a vacuum rotary evaporation for further determination. The experimental groups were named after the type and order of enzymes used in each group, listed as P (single protease hydrolysis), C (single chitinase hydrolysis), CP (simultaneous hydrolysis using protease and chitinase), P/C (two-step hydrolysis using protease followed by chitinase) and C/P (two-step hydrolysis using chitinase followed by protease). Each group was compared with the control experiment in the absence of enzymes under the same conditions. After enzymolysis, the micrographs of Antarctic krill residuals in each group were analyzed with an inverted fluorescence microscope (IX83, Olympus, Tokyo, Japan).

### 3.3. Extraction of Antarctic Krill Oil

With anhydrous ethanol as extraction solvent, the experiment was carried out as follows [10]: The sediment obtained from enzymolysis was added into ethanol (1/5, *w*/*v*) and stirred with an overhead stirrer (RW20DZM, IKA, Guangzhou, China) at 150 rpm at room temperature for 120 min. After extraction, the mixture was centrifuged at 3400× *g* for 20 min to separate the oil-rich organic phase. Then, a secondary centrifugation was conducted at 10,000× *g* for 20 min for further refining. The supernatant and precipitate obtained after the second centrifugation was then collected. The solvent was removed at 35 °C in a vacuum rotary evaporation. The precipitate was dried at 50 °C in an oven to constant weight that was defined as the impurity. The smashed Antarctic krill without enzymolysis were centrifuged at 3400× *g* for 20 min to remove water and extracted at the same condition as control.

In contrast, the extraction of AKO was also conducted using conventional Soxhlet apparatus with ethanol as the solvent. According to the method of Kim et al. [35], 10 g of smashed Antarctic krill and 200 mL of ethanol were added into the Soxhlet apparatus for lipid extraction at 120 °C for 8 h.

### 3.4. Optimization of Lipid Yield

Under the pH and temperature conditions in Section 3.2, single-factor experiments were conducted to optimize the lipid yield of the two-step chitinase/protease hydrolysis by prolonging the enzymolysis time. Firstly, the hydrolysis time of alkaline protease was fixed (4 h), and the hydrolysis time of chitinase was set as 2, 4, 6, 8, 10, 12 and 14 h. The lipid yield was analyzed to determine the optimal hydrolysis time of chitinase. Then, the optimal chitinase hydrolysis time was fixed, and the alkaline protease treatment time was set as 2, 4, 6 and 8 h. The AKO yield was analyzed, and the optimal alkaline protease hydrolysis time was determined.

### 3.5. Determination of Lipid Yield and Impurity Content

The amount of the initial Antarctic krill meal and the amount of the AKO and impurity obtained in each group were recorded. The lipid yield was calculated as follows [1]:

Lipid yield (%) = a/m × 100,(1)

where a is the weight in grams of the AKO, m is the mass in grams of the initial Antarctic krill meal.

The impurity content of the crude AKO was calculated as follows:

Impurity content (%) = b/(a + b) × 100,(2)

where a is the weight in grams of the AKO, b is the weight in grams of the impurity.

### 3.6. Determination of Antioxidant Capacity

The antioxidant capacity of AKO and enzymolysate was determined using DPPH and ABTS radical scavenging methods described by Xie et al. [1] and Szydłowska-Czerniak and Baszewska [36] with slight modifications. In brief, 0.3 g of AKO or enzymolysate was dissolved in 50 mL of ethanol or water, respectively. For DPPH assay, 2 mL of sample solution was added to 2 mL of 0.17 mmol/L DPPH• solution and thoroughly mixed. After being incubated in darkness for 30 min, the absorbance of the mixture was determined at 517 nm using a UV–visible spectroscopy system (UV5100, Metash, Shanghai, China). Then, 2 mL of ethanol or water was added to 2 mL of DPPH• solution as negative control for AKO or enzymolysate, respectively. For each sample, 2 mL of sample solution was added to 2 mL of ethanol as blank control.

For ABTS•^+^ scavenging activity, 7 mmol/L ABTS solution was mixed with 2.45 mmol/L potassium persulfate solution (2/1, *v*/*v*) and kept in darkness for 16 h. The obtained ABTS•^+^ solution was diluted 12 times with ethanol or water for further AKO or enzymolysate analysis, respectively. Then, 1 mL of sample solution was mixed with 2 mL of diluted ABTS•^+^ solution and incubated at 30 °C for 5 min. After that, the absorbance was measured at 734 nm. Similarly, a negative control and a blank control were determined at the same time.

The free radical inhibition was calculated as follows:

Inhibition (%) = [1 − (A_1_ − A_2_)/A_0_] × 100, (3)

where A_1_ is the absorbance of a test solution, A_2_ is the absorbance of a blank solution, and A_0_ is the absorbance of negative control.

Trolox was analyzed as a standard antioxidant and its standard curves of the radical inhibition were drawn. The trolox equivalent (TE) for AKO or enzymolysate was calculated and reported in mmol TE/100 g sample.

### 3.7. Analytical Methods

#### 3.7.1. Determination of Phospholipid

The PL content was analyzed with an HPLC (1525-2707, Waters, Milford, CT, USA) equipped with an evaporative light scattering detector (2424, Waters, Milford, MA, USA) and a SinoChrom Si60 column (250 mm × 4.6 mm, 5 µm, Elite, Dalian, China). The mobile phase A was n-hexane/isopropanol/13% acetic acid solution (4/5/1, *v*/*v*/*v*) containing 0.016% (*v*/*v*) triethylamine, and mobile phase B was n-hexane/isopropanol (4/6, *v*/*v*) containing 0.016% (*v*/*v*) triethylamine. A certain amount of AKO sample was dissolved in a chloroform/methanol mixed solvent (2/1, *v*/*v*) and filtered. The flow rate was 1 mL/min. The gradient elution condition was as follows: 0–15 min, 70–0% B (*v*/*v*); 15–22 min, 100% A (*v*/*v*). The samples were detected with a high purity nitrogen pressure at 30 psi, spray temperature of 36 °C and drift tube temperature of 60 °C. The total PL content of the injected AKO sample was the sum of PC and PE contents calculated according to the corresponding standard curve and reported in mg/g oil. The PL yield was reported in mg/g krill.

#### 3.7.2. Determination of Free EPA and DHA

An HPLC equipped with an evaporative light scattering detector was used to determinate F-EPA and F-DHA [10]. The column was Supersil ODS2 (250 mm × 4.6 mm, 5 µm, Elite, Dalian, China) and the AKO sample was resuspended in methanol and filtered before injection. The F-EPA and F-DHA contents of the injected AKO sample were calculated according to the corresponding standard curve and reported in mg/g oil. Their yields were reported in mg/g krill.

#### 3.7.3. Determination of Astaxanthin

The astaxanthin content was detected using a method developed by Zeb et al. with slight modifications [37]. An HPLC equipped with an ultraviolet detector (2487, Waters, Milford, MA, USA) and a C18 column (250 mm × 4.6 mm, 5 µm, Elite, Dalian, China) was conducted. The mobile phase A was methanol, and mobile phase B was acetonitrile. Isometric elution was performed at 5% A and 95% B (*v*/*v*) at a flow rate of 1 mL/min. The detection wavelength was 476 nm. The astaxanthin content of the injected AKO sample was calculated and reported in mg/g oil. Astaxanthin yield was reported in mg/g krill.

#### 3.7.4. Determination of Protein Content and Composition

The soluble protein content of the enzymolysate was analyzed by spectrophotometry [38]. BCA protein assay kit was used to detect the absorbance value at 562 nm with BSA as the standard.

Suspended polypeptides obtained after precipitation with trichloroacetic acid were assayed according to the method of Lowry et al. [39] to measure the oligopeptide content in the enzymolysate. The diluted samples and BSA (as the standard) were assayed simultaneously at 500 nm.

Amino acid content was analyzed by the Ninhydrin method described by Jain and Badve [40] with slight modifications using l-glutamic acid as the standard. In detail, the mixture of ninhydrin reagent, phosphate buffer and diluted sample was incubated at 100 °C for 15 min and the absorbance at 570 nm was measured after cooling. The contents of water-soluble proteins, polypeptides and amino acids in the enzymolysate were calculated and reported in mg/g krill.

#### 3.7.5. Determination of Carbohydrate Content and Composition

Using glucose as the standard, the polysaccharide content of the enzymolysate was measured by the phenol–sulfuric acid method established by Dubois et al. [41]. The diluted samples were mixed with phenol and sulfuric acid, and it reacted at room temperature for 15 min. The absorbance at 490 nm was measured.

The reducing sugar content of the enzymolysate was measured by the 3,5-dinitrosalicylic acid (DNS), reducing sugar assay described by Miller [42]. In brief, the prepared DNS was added to the diluted samples and incubated at a boiling water bath for 5 min. After cooling, the absorbance at 540 nm was measured. The glucose was analyzed simultaneously as the standard. The contents of polysaccharides and reducing sugars in the enzymolysates were calculated and reported in mg/g krill.

### 3.8. Statistical Analysis

All experimental data were derived from three independent replicates unless otherwise mentioned. Statistical analysis was conducted by one-way analysis of variance (ANOVA), and the significance of each mean value was determined (*p* < 0.05) with the Duncan’s multiple range tests and the independent samples *t* test using the SPSS software (SPSS Inc., Chicago, IL, USA).

## 4. Conclusions

This study provided a novel efficient extraction of AKO and enzymolysate based on two-step enzymolysis. By comparing the oil yield, the sequence of enzymolysis was determined, i.e., chitinase followed by protease, and the enzymolysis time was then optimized. Using two-step enzymolysis-assisted ethanol extraction, the lipid yield of AKO was 4.18%, which was 112% and 200% of Soxhlet extraction and the control under the same solvent and extraction conditions, respectively. In addition, the two-step enzymolysis-assisted extraction could significantly reduce the impurity content and effectively increase the PL, FFA and astaxanthin yields. Furthermore, the enzymolysate as by-product contained abundant nutrient components, e.g., soluble peptides, amino acids, oligosaccharides, and N-acetyl-D-glucosamine. Both AKO and enzymolysate showed high DPPH• and ABTS•^+^ scavenging ability. These results showed that the two-step enzymolysis-assisted extraction could obtain high-quality AKO with high yield, which provided a new approach for simultaneous production of AKO and enzymolysate with high antioxidant capacity in the krill industry.

## Figures and Tables

**Table 1:** Active components of Antarctic krill oil extracted under the optimal conditions.

Active Components	Ethanol Extraction (Control) ^1^	Two-Step Enzymolysis Extraction ^2^
PL (mg/g oil)		
PC	269.60 ± 2.64 ^a^	219.14 ± 8.59 ^a^
PE	31.75 ± 0.78 ^b^	26.91 ± 0.11 ^a^
FFA (mg/g oil)		
F-EPA	24.88 ± 0.29 ^a^	46.58 ± 0.08 ^b^
F-DHA	23.15 ± 0.84 ^a^	34.38 ± 0.64 ^b^
Astaxanthin (mg/g oil)	0.96 ± 0.02 ^b^	0.82 ±0.01 ^a^

^1&2^ Different superscript letters in a row indicate significant differences for an active component (independent samples *t* test, *p* < 0.05).

**Table 2:** Nutritional components of the enzymolysate extracted under the optimal conditions.

Active Components	First Enzymolysate ^1^	Second Enzymolysate ^2^	Total
Proteins (mg/g krill)			82.52 ± 2.08
Soluble proteins	21.32 ± 0.86 ^b^	13.03 ± 0.20 ^a^	34.35 ± 0.79
Oligopeptides	8.05 ± 0.24 ^b^	5.87 ± 0.17 ^a^	13.92 ± 0.40
Amino acids	21.94 ± 0.65 ^b^	12.30 ± 0.99 ^a^	34.24 ± 0.89
Carbohydrates (mg/g krill)			5.79 ± 0.05
Polysaccharides	3.72 ± 0.05 ^b^	1.43 ± 0.06 ^a^	5.16 ± 0.04
Reducing sugars	0.32 ± 0.01 ^a^	0.31 ± 0.00 ^a^	0.63 ± 0.00

^1&2^ Different superscript letters in a row indicate significant differences for an active component (independent samples *t* test, *p* < 0.05).

## Data Availability

The data that support the findings of this study are available from the corresponding author, Zhi-Long Xiu, upon reasonable request.
